# 

**Published:** 1855-10

**Authors:** 


### THE WESTERN JOURNAL OF MEDICINE AND SURGERY.

NEW SERIES. OCTOBER, 1855. VOL. IV.—NO. 10.

EDITED BY
LUNSFORD P. YANDELL, M. D.
PROFESSOR OF PHYSIOLOGY AND PATHOLOGICAL ANATOMY, IN THE UNIVERSITY OF LOUISVILLE.

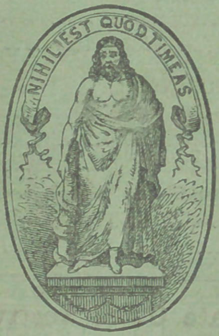

LOUISVILLE, KY:
PRINTED AND PUBLISHED BY J. F. BRENNAN,
*Cor. of Market & First Streets.*

☞PRICE, $3 A YEAR—INVARIABLY IN ADVANCE.—POSTAGE, 2 CENTS A NUMBER.☜

